# Efficacy of Adaptive Biofeedback Training in Treating Constipation-Related Symptoms

**DOI:** 10.1155/2015/959734

**Published:** 2015-05-03

**Authors:** Jing Tang, Zhihui Huang, Yan Tan, Nina Zhang, Anping Tan, Jun Chen, Jianfeng Chen

**Affiliations:** ^1^Division of Gastroenterology, Affiliated Hospital of Hainan Medical College, Haikou 571000, China; ^2^Ningbo Pace Translational Medical Research Center, Beilun, Ningbo 315000, China; ^3^Department of Gastroenterology, Sir Run Run Shaw Hospital, School of Medicine, Zhejiang University, Hangzhou 310000, China; ^4^Divison of Gastroenterology, The First Affiliated Hospital of Nanjing Medical University, Nanjing 210000, China; ^5^Ningbo Medkinetic Inc., Ningbo 315000, China

## Abstract

Biofeedback therapy is a well-known and effective therapeutic treatment for constipation. A previous study suggested that adaptive biofeedback (ABF) training was more effective than traditional (fixed training parameters) biofeedback training. The aim of this study was to verify the effectiveness of ABF in relieving constipation-related symptoms. We noticed that in traditional biofeedback training, a patient usually receives the training twice per week. The long training sessions usually led to poor compliance. This study proposes an intensive biofeedback therapy and compares intensive therapy with nonintensive therapy in patients with constipation-related symptoms. *Methods.* 63 patients with constipation-related symptoms were treated with ABF between 2012 and 2013. These patients were further divided into the intensive therapy and nonintensive therapy groups. *Results.* A total of 63 patients were enrolled in the study, including 24 in the nonintensive therapy group and 39 in the intensive therapy group. 100% (*N* = 21) of constipation patients achieved the primary efficacy endpoint (≥3 bowel movements/week). There was significant improvement in constipation-related symptoms after adaptive biofeedback. The intensive biofeedback therapy did not show better performance compared to nonintensive biofeedback therapy. *Conclusions.* This investigation provides support for the efficacy of biofeedback for constipation-related symptoms. The efficacy of intensive therapy is similar to nonintensive therapy.

## 1. Introduction

Chronic constipation is a common disorder characterized by defecation difficulty or decreased bowel movements (less than three times a week). The worldwide prevalence of chronic constipation varies from 12% to 17% [1]. It is more prevalent in females than males (prevalence rate of 2.2 : 1) and the prevalence increases with age [2]. Patients who reported persistent constipation have decreased health-related quality of life and higher level of depression [3]. Chronic constipation has a great economic and social impact, including laboratory tests, diagnostic procedures, and healthcare expenditures [4].

Constipation is primarily a functional disorder, and it could also be caused by medications, diseases of the colon, metabolic disturbances, and neurologic disorders. Constipation can be categorized into 3 subgroups (obstructed defecation, slow transit constipation, and normal transit constipation) [5, 6]. About 40% of constipation is due to obstructed defecation [7, 8]. Obstructed defecation (also known as dyssynergic defecation, pelvic floor dyssynergia, or outlet obstruction) is characterized by the lack of coordination between the abdominal and pelvic floor muscles during defecation. Obstructed defecation is caused by one of the following problems: impaired rectal contraction, paradoxical anal contraction, or inadequate anal relaxation.

Although currently available treatment options have been reported to be effective at improving patients' symptoms, the curative effect is still unsatisfactory. There is insufficient data to support that lifestyle and diet change such as increased fiber and fluid intake can improve chronic constipation. Laxatives (including bulking agents, osmotic and stimulant laxatives, and stool softeners) have been approved to relieve the symptoms [9–11]. However, laxatives do not target the underlying pathophysiology, such as paradoxical anal contraction. Biofeedback therapy, an instrument-based learning process, can correct the incoordination of the abdominal, rectal, and anal sphincter pressures [12]. The efficacy of biofeedback therapy is reported to range from 44% to 100% in various clinical studies [13]. However, training requires complex processing and the training targets are fixed, meaning all patients receive the same training regardless of different anorectal motility and ability to achieve the training goal. A novel method of adaptive biofeedback (ABF) training reportedly changes the training targets and protocols according to patients' anorectal motility. This method of ABF has shown to be superior to the traditional biofeedback training [14].

The frequency and duration of traditional biofeedback training are variable in different clinical trials [15–18]. On average, patients are asked to receive treatment for 3 months at a frequency of twice per week. The inconvenience and lengthy duration of biofeedback treatment often lead to poor compliance. We propose an intensive biofeedback therapy once a day or once every other day. The aim of the present study was to confirm the efficacy of ABF and compare the efficacy of intensive therapy with nonintensive therapy in patients with constipation-related symptoms.

## 2. Materials and Methods

A retrospective cohort study was conducted on subjects who had been treated with ABF for constipation-related symptoms between April 2012 and September 2013. The results were compared between the intensive therapy and nonintensive therapy in terms of constipation-related symptoms. The subjects were selected in this study according to the following inclusion/exclusion criteria.

### 2.1. Inclusion and Exclusion Criteria

The study enrolled men and women, aged ≥ 18 years, with a history of constipation-related symptoms. Constipation-related symptoms are defined as follows: <3 bowel movements (BMs) per week on average, hard stools, low stool volume, sensation of incomplete evacuation, straining at defecation, or a need for manual maneuver to facilitate evacuation. Exclusion criteria included drug-induced constipation, metabolic, endocrine, neurological disorders, surgical obstruction, megacolon/megarectum, surgical obstruction, and pseudoobstruction. Other exclusion criteria were severe cardiovascular, renal, liver, or lung diseases.

### 2.2. Outcomes and Data Collection

#### 2.2.1. Primary Outcomes

Patients rate the severity of constipation in terms of bowel movements with the three-point scale classification [0 = normal (≥3 BMs per week), 1 = mild (1-2 BMs per week), 2 = severe (<1 BMs per week)]. Criteria for therapeutic effects are being cured (BMs changed from severe or mild to normal), being effective (BMs changed from severe to mild), and having no effect (BMs did not change).

#### 2.2.2. Secondary Outcomes

Secondary outcome measures usage of medications, defecation difficulty, hard stools, straining, incomplete bowel movement, low stool volume, manual maneuver to facilitate, abdominal bloating, and anus discomfort. Symptoms of defecation difficulty, hard stools, incomplete bowel movement, low stool volume are described on a 0–3 scale (0 = absent, 1 = mild, 2 = moderate, 3 = severe), manual maneuver to facilitate [0 = absent, 1 = mild (<1 time per week), 2 = moderate (1–3 times per week), 3 = severe (>3 times per week)].

#### 2.2.3. Impact on Social Activities and Work

The impact on social activities and work is rated on a 0–2 scale where 0 = absent, 1 = mild (a mild effect on normal social activities and normal work), and 2 = severe (a severe effect). Criteria for therapeutic effects are being cured (change from severe or mild to absent), being effective (change from severe to mild), and having no effect (no change).

### 2.3. Adaptive Biofeedback Training

Biofeedback training for the treatment of constipation is to train the relaxation of anal sphincter, enhance the sensory perception, and improve the rectoanal coordination. Training of rectoanal coordination is to increase the pushing effort as reflected by an increase in intra-abdominal/intrarectal pressures and synchronized relaxation reflected by a decrease in anal sphincter pressure. However, the traditional biofeedback training algorithm uses the fixed training target, it cannot increase (or decrease) the training strength or duration based on patient's capacity. On the other hand, the adaptive biofeedback training (ABT) (Ningbo Maida Medical Device Inc. Ningbo, China.) method uses the training strength and duration based on patient's own capacity and trains the patient at strength slightly above his or her own threshold with the purpose to gradually increase the strength threshold until the targeted threshold is met. It was reported to have a better efficacy for the treatment of constipation than the traditional biofeedback training method. Each patient received a total of 16 training sessions with each training session lasting half an hour.

*Intensive Therapy*. Patients were asked to receive intensive biofeedback therapy once a day or once every other day.

*Nonintensive Therapy*. Patients received nonintensive training twice a week in the motility lab.

### 2.4. Statistical Analysis

The data are expressed as mean ± standard error. The paired-sample* t*-test was used to compare defecation difficulty, hard stools, straining, incomplete bowel movement, low stool volume, manual maneuver to facilitate, abdominal bloating, and anus discomfort before and after treatment with ABF. An independent* t*-test was used to compare the nonintensive therapy with the intensive therapy group. Data were considered statistically significant if *P* < 0.05.

## 3. Result

A total of 63 subjects met the inclusive criteria. 21 subjects had a long history of constipation defined as an average of <3 BMs per week. The mean age of the participants was 45.60 ± 16.60 and 42 (66.66%) were women and 21 were men. There was no significant difference in age and gender between the two treatment groups.

**Figure 1:**
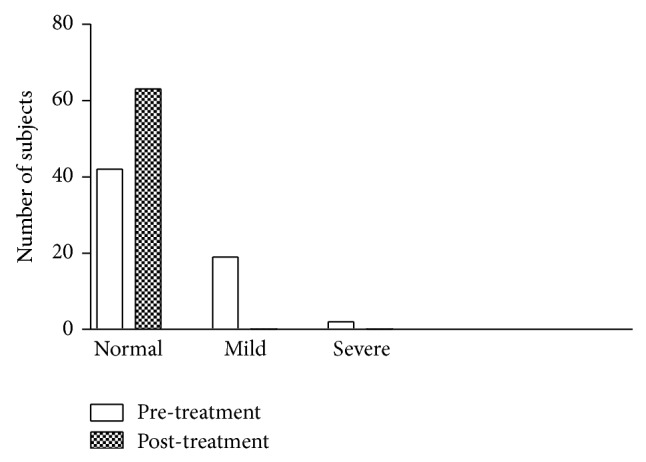
Effects of ABF on bowel movement (BM).

**Figure 2:**
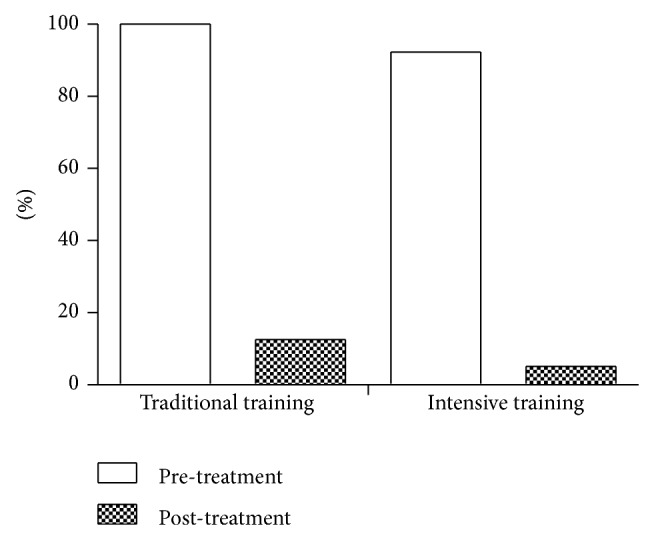
Usage of medications during the biofeedback training.

After adaptive biofeedback training treatment, all constipation patients (*N* = 21) reported a significantly greater number of weekly bowel movements (≥3 times) compared with the baseline (<3 times). The cure rate of nonintensive therapy (*N* = 8) and intensive therapy (*N* = 13) both reached 100%. None of the patients reported less than 3 BMs per week after the treatment (Figure 1). The usage of medications decreased considerably during the training period in both treatment groups compared to baseline. The medication usage at the start of treatment was 100% for nonintensive therapy group and 92.3% for intensive therapy group. During the treatment period, medication usage decreased to 12.5% for the nonintensive therapy group and 5.1% for the intensive therapy group (Figure 2).

As shown in Table 1, defecation difficulty, hard stools, and straining significantly improved with nonintensive therapy/intensive therapy compared with baseline (*P* < 0.05). Intensive therapy patients also reported significant improvements in incomplete BM. Intensive therapy also improved low stool volume (*P* = 0.006) and decreased manual maneuver frequency (*P* = 0.048). Both treatments significantly decreased abdominal bloating (*P* < 0.05). Nonintensive therapy, but not intensive therapy, significantly reduced the scores for anus discomfort (0 versus 0.48 + 0.87, *P* = 0.011; 0 versus 0.10 + 0.50, *P* = 0.21). However, there was no statistically difference between the two methods in all symptoms (*P* > 0.05).

**Figure 3:**
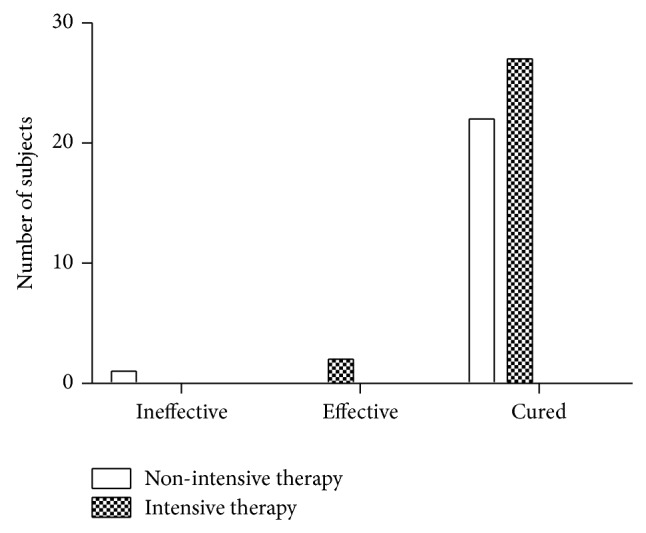
Improve the impact on social activities and work.

Overall, 82.5% (*N* = 52) of subjects reported that constipation symptoms interfered with normal social activities and normal work. The number of patients receiving either nonintensive therapy or intensive therapy who were cured was high (22 and 27, resp.). Only 1 patient with nonintensive therapy showed no improvement (Figure 3).

## 4. Discussion

The results of this study indicate that adaptive biofeedback training was effective in the treatment of patients with constipation-related symptoms. The adaptive biofeedback training was able to significantly increase weekly bowel movements. Patients also showed major improvement in defecation difficulty, hard stools and straining, incomplete BM, low stool volume, manual maneuver to facilitate, and abdominal bloating. In the current study, adaptive biofeedback training also reduced the impact on social activities and work created by constipation-related symptoms.

Our results are consistent with the study conducted by Xu et al. [14] who recently reported that adaptive biofeedback training was more effective in improving bowel movements than those of conventional fixed biofeedback training (3.4 ± 1.3 versus 2.6 ± 0.5, *P* < 0.005). In this study, twenty-one constipation patients (100%) had bowel movements of more than 3 times per week after ABF therapy. Chiarioni et al. [15] reported 82% of patients had ≥3 bowel movements per week at 12-month follow-up after fixed biofeedback training. Only 29% patients reported ≥3 bowel movements per week at 4 weeks of prucalopride therapy [19]. The ABF had a higher bowel movement response rate than fixed biofeedback training and laxative.

ABF significantly improved symptoms of constipation, such as defecation difficulty, incomplete BM, hard stools, and straining based on ROME III criteria [20]. Xu et al. [14] reported that ABF significantly improved these symptoms compared with fixed biofeedback training.

In addition, the impact of constipation symptoms on social activities and work was significantly decreased at the end of ABF. A growing evidence shows that constipation patients have a significantly impaired health-related quality of life compared with population norms [21–23]. Although this study did not use standard assessment tools to characterize quality of life, the results indicated that symptoms had an impact on social function. Other studies reported that fixed biofeedback training improved the quality of life scores compared with control group [18, 24].

In this study, we investigated the efficacy of intensive therapy compared to nonintensive therapy. In previous studies, patients were asked to receive nonintensive biofeedback training twice a week with a total of 4 to 6 sessions [25]. We proposed an intensive biofeedback therapy of which frequency was once a day or once every other day. There was no significant difference in constipation-related symptoms between the two treatment groups. Several randomized controlled trials had variable duration and number of biofeedback sessions, but the efficacy of therapy was similar [15–18, 26]. But the intensive biofeedback therapy had short duration and may have better compliance.

In conclusion, treatment with adaptive biofeedback training produced significant improvement in bowel movements. ABF also significantly improved symptoms associated with constipation. The intensive biofeedback therapy did not seem to be superior to nonintensive therapy.

## Figures and Tables

**Table 1:** Constipation-related symptoms before and after intensive therapy/nonintensive therapy.

	Intensive therapy		Nonintensive therapy	
	Before training	After training	Before training	After training
Defecation difficulty	1.18 + 1.12	0.13 + 0.41^∗^	0.79 + 1.06	0.17 + 0.48^∗^
Staining	0.44 + 0.97	0.05 + 0.22^∗^	0.58 + 0.93	0^*^
Incomplete BM	0.41 + 0.82	0.03 + 0.16^∗^	0.25 + 0.68	0
Low stool volume	0.67 + 1.01	0.26 + 0.50^∗^	0.17 + 0.57	0.04 + 0.20
Hard stools	0.67 + 1.06	0.10 + 0.31^∗^	1.04 + 1.08	0.13 + 0.45^∗^
Manual maneuver to facilitate	0.23 + 0.71	0^*^	0.08 + 0.41	0
Abdominal bloating	0.46 + 0.88	0.03 + 0.16^∗^	0.96 + 1.20	0.04 + 0.20^∗^
Anus discomfort	0.10 + 0.50	0	0.50 + 0.89	0^*^

^∗^ *P* < 0.05 versus before training.
